# Comparing clinical features of behavioral variant frontotemporal dementia and Alzheimer's disease using network analysis

**DOI:** 10.1002/alz.70361

**Published:** 2025-06-17

**Authors:** Grace J. Goodwin, Sebastian Mehrzad, Jeffrey L. Cummings, Brenna N. Renn, Jefferson W. Kinney, Samantha E. John

**Affiliations:** ^1^ Department of Psychology University of Nevada Las Vegas Nevada USA; ^2^ Department of Brain Health Kirk Kerkorian School of Medicine University of Nevada Las Vegas Nevada USA; ^3^ Princeton Neuroscience Institute Princeton University Princeton New Jersey USA; ^4^ Chambers‐Grundy Center for Transformative Neuroscience Department of Brain Health Kirk Kerkorian School of Medicine University of Nevada Las Vegas Nevada USA

**Keywords:** Alzheimer's disease, behavioral variant frontotemporal dementia, cognitive, mild cognitive impairment, neuropsychiatric, neuropsychology

## Abstract

**INTRODUCTION:**

Clinical characterization of behavioral variant frontotemporal dementia (bvFTD) and Alzheimer's disease (AD) is challenging due to overlapping neuropsychiatric symptoms and cognitive profiles between the two conditions.

**METHODS:**

We used clinical network analysis to characterize and compare clinical profiles in AD and bvFTD using initial visit data from the National Alzheimer's Coordinating Center.

**RESULTS:**

The final matched sample included 890 patients per group (AD: mean age * *= 63.02, standard deviation [SD] = 9.34, 36.4% female; bvFTD: mean age * *= 62.87, SD = 9.46, 36.52% female). Both networks were densely connected, reflecting comorbidity between neuropsychiatric symptoms and cognitive scores. Memory performance, hallucinations, and motor disturbance were bridge symptoms in the AD network, whereas elation was the sole bridge symptom in the bvFTD network.

**DISCUSSION:**

Distinct networks highlight unique clinical profiles in AD and bvFTD. Treatment of bridge symptoms may relieve overall symptom burden. Findings can advance clinical characterization of AD and bvFTD, leading to development of targeted interventions.

**Highlights:**

- We compared clinical features of Alzheimer's disease (AD) and behavioral variant frontotemporal dementia (bvFTD).
- Clinical networks showed comorbidity between neuropsychiatric symptoms and cognitive manifestations.
- Clinical networks significantly differed between AD and bvFTD, highlighting unique behavioral and cognitive profiles.
- Distinct symptoms were important for overall symptom comorbidity.
- Findings can be used to characterize AD and bvFTD and inform targeted treatment.

## 1 BACKGROUND

Alzheimer's disease and related dementias (ADRD) are increasingly common among older adults. Alzheimer's disease (AD) is the most common cause of dementia,^1^ and frontotemporal dementia (FTD) syndromes (e.g., behavioral variant frontotemporal dementia [bvFTD]) are the second leading cause of early‐onset dementia.^2^ ADRD are characterized by cognitive decline (COG) as well as neuropsychiatric symptoms (NPS), which are distressing for patients and caregivers and are associated with accelerated decline in independence,^3^ greater caregiver burden, and increased risk for hospitalization or admission to skilled nursing facilities.^4^, ^5^ Accurate clinical characterization of ADRD is essential for access to disease‐modifying and symptom‐reducing treatments that can delay clinical progression, functional decline, admission to long‐term care, and dependence on caregivers.^4^, ^5^

Distinction of AD and bvFTD has improved with clinically validated AD biomarkers,^6^, ^7^ but biomarker analyses are not readily accessible across all settings. Aspects of syndrome assessment are more accessible to patients and caregivers compared to costly and highly specialized biomarker evaluation. Currently, self or informant report of symptoms and objective neuropsychological testing is essential to the characterization of bvFTD and AD.^8^, ^9^ However, clinical definitions of bvFTD and AD have received criticism due to overlapping symptoms.^10^, ^11^ Several clinical features are common in both AD and bvFTD, whereas others are preferentially, although not exclusively, associated with each syndrome.^12^, ^13^ Although these commonalities can complicate diagnosis,^14^ they can also signal possible shared neural mechanisms, which may be useful for the development of treatment targets. Innovative applications of clinical data are necessary for clarifying clinical phenotypic signatures of bvFTD and AD.

Despite evidence of covariation between COG and NPS in ADRD,^4^, ^15^, ^16^ existing assessment approaches do not consider covariation among these domains. Network analysis, a psychometric approach for describing syndrome structures, can advance understanding of common and distinct clinical mechanisms in bvFTD and AD. Using this approach, syndromes are viewed as a set of mutually reinforcing and co‐occurring clinical features^17^, ^18^, ^19^ interacting within and between domains of functioning. Clinical networks can identify “influential symptoms” that serve as bridge connections between symptom communities (e.g., NPS community and COG community),^20^ and targeting bridge symptoms can ameliorate comorbidity and overall symptom burden.^18^ Clinical network analysis has been used to characterize symptoms in AD^21^, ^22^ as well as other neuropsychiatric syndromes^23^, ^24^ and has been useful for identifying potential intervention targets in these populations. Similarly, modeling and comparing clinical networks in bvFTD and AD may advance syndrome characterization and improve understanding of shared clinical mechanisms.

The current study used the National Alzheimer's Coordinating Center (NACC) Uniform Data Set (UDS) to characterize complex symptom relationships in bvFTD and AD at initial NACC visit. Separate clinical networks were estimated for the bvFTD and AD groups, and bridge symptoms were identified to determine the symptoms responsible for comorbidity of NPS and COG. We expected networks to differ significantly between bvFTD and AD, implicating distinct comorbidity patterns. This study aims to demonstrate the utility of network analysis in the clinical characterization of ADRD syndromes.

## 2 METHODS

### 2.1 NACC data characteristics

NACC provides a comprehensive data repository for research on neurodegenerative disorders, including AD and bvFTD. NACC utilizes a UDS that contains longitudinal data that have been collected since 2005, at National Institute on Aging (NIA)–funded Alzheimer's Disease Research Centers (ADRCs) across the United States. Data elements and collection methods have been described previously.^25^, ^26^, ^27^, ^28^ The NACC UDS includes demographic, neuropsychological, behavioral, medical, and health history data in order to accurately diagnose neurodegenerative diseases and track their course.^28^ Participants and study partners enrolled at each ADRC provide written consent as part of the institutional review board (IRB)–approved protocol at that site. This consent covers both the data collection procedures required by the respective center as well as the inclusion of the participant's data in the larger NACC UDS database. Relevant to this study, NACC is the only database offering a large sample of patients with comprehensive demographic, clinical, genetic, neuropsychiatric, and neurocognitive data.

### 2.2 Participants and sample selection

Participants were selected from the NACC UDS (all versions; (https://naccdata.org/). Patient evaluations were completed at funded ADRCs during the period between September 2005 and the freeze date of June 2023; data from participants’ initial NACC visit were used (*N* = 48,605 with initial visit data). We used the following criteria for AD sample identification: AD was the primary or contributing cause of observed impairment; no comorbid diagnosis of bvFTD, primary progressive aphasia (PPA), progressive supranuclear palsy (PSP), corticobasal syndrome (CBS), dementia with Lewy bodies (DLB), or vascular dementia (VaD); no evidence of early‐onset AD (e.g., presenilin‐1 [PSEN‐1], PSEN‐2, or amyloid precursor protein [APP]). Biomarker confirmation was not used for AD sample characterization due to limited sample availability. We used the following criteria for bvFTD sample identification: bvFTD was the primary or contributing cause of observed impairment; no comorbid diagnosis of AD, PPA, PSP, CBS, DLB, or VaD. Groups were propensity score matched on age, sex, education level, and ethnicity/race using the *MatchIt* package.^29^

### 2.3 Measures

#### 2.3.1 Participant characterization

Participants were described using multiple clinical and diagnostic variables in the UDS. Level of cognitive impairment was identified from a NACC‐derived diagnostic severity (e.g., mild cognitive impairment [MCI] or dementia) variable.^30^ Participants with a cognitive status of MCI or dementia were included in the sample (e.g., cognitive status at visit = 3 [MCI] or 4 [dementia]).^27^, ^31^ Etiological diagnosis was identified from NACC‐derived diagnosis variables that specify whether the etiological diagnosis is a primary or contributing cause of observed impairment.^28^ Participants are also described through the Clinical Dementia Rating (CDR©) dementia global staging instrument, a 5‐point scale that characterizes six domains of cognitive and functional abilities.^32^ Information is obtained through a semi‐structured interview of the patient and informant, and clinicians rate the patient's level of overall impairment on a 5‐point scale (0.0 = *No impairment;* 0.5 = *Questionable impairment*; 1.0 = *Mild impairment*; 2.0 *Moderate impairment*; and 3.0 = *Severe impairment*).

**RESEARCH IN CONTEXT**

1. **Systematic review**: Clinical symptoms (neuropsychiatric symptoms and cognitive deficits) are characteristic of Alzheimer's disease (AD) and behavioral variant frontotemporal dementia (bvFTD). The extensive literature has noted difficulties with accurate clinical characterization of AD and bvFTD using existing criteria. Authors used PubMed to identify research comparing clinical functioning in AD and bvFTD, and relevant publications are referenced throughout.
2. **Interpretation**: We provide evidence of distinct clinical networks between AD and bvFTD using network analysis. Treatment of memory deficits, hallucinations, and motor disturbance in AD and elation in bvFTD may result in overall symptom improvement.
3. **Future directions**: Future research simulating treatment of clinical networks may provide a precise approach to clinical trial design and intervention development. Validation studies, using pathologically confirmed cases of AD and bvFTD, are needed to determine the utility of clinical symptoms for differential diagnosis and treatment targets.

#### 2.3.2 Primary outcome measures

##### Neuropsychiatric symptoms

The Neuropsychiatric Inventory – Questionnaire (NPI‐Q) is a widely used measure to assess NPS in clinical and research settings.^33^ The NPI‐Q relies on a caregiver/informant report of the presence and severity of 12 NPS within the past month. Assessed symptoms include delusions, hallucinations, agitation/aggression, depression/dysphoria, anxiety, elation/euphoria, apathy/indifference, disinhibition, irritability/lability, motor disturbance, nighttime behaviors, and appetite/eating problems.^33^ NPI‐Q symptom presence (0* = symptom not endorsed*, 1* = symptom endorsed*) and severity (1 = *mild*, 2 = *moderate*, 3 = *severe*) variables were combined to create one severity variable per symptom (0 = *symptom not endorsed*, 1 = *mild*, 2 = *moderate*, 3 = *severe*). The NPI‐Q has satisfactory psychometric properties, including acceptable test–retest reliability and convergent validity.^33^

##### Neurocognitive functioning

The NACC neuropsychological test battery provides standardized and comprehensive measures of neurocognitive function across several domains, including verbal episodic memory, attention, language, psychomotor speed, and executive function.^28^ Raw test scores are available for each neurocognitive subtest at each assessment time[Fig alz70361-fig-0001] point. Our model includes performance scores from the participant's first visit for eight subtests assessing several cognitive domains. Category fluency is a measure of speeded semantic fluency, where participants are asked to name exemplars of a given semantic category and the number of unique correct responses provided within the time limit are scored.^34^ Category fluency scores represent the total score on two semantic categories. Trail Making Test (TMT) Parts A and B are measures of cognitive processing speed and set‐shifting, respectively. Participants are asked to connect numbers and letters by drawing lines in number order (TMT‐A) and alphanumeric order (TMT‐B), and total time to complete the task is scored.^35^ The Wechsler Adult Intelligence Scale – Revised (WAIS‐R) Digit Symbol Substitution Test is a measure of cognitive processing speed where participants must match symbols with their corresponding numbers under a time limit. Total correct responses are scored, with higher scores representing more items completed.^36^ The Boston Naming Test (BNT)^37^ and Multilingual Naming Test (MINT)^27^ measure object recognition and confrontation naming. The total number of correctly identified items is scored, with higher scores representing better performance. Wechsler Memory Scale – Revised (WMS‐R) Logical Memory IIA‐Delayed Recall^38^ and Craft Story‐21 Recall‐Delayed^27^ measure delayed recall. Total number of correctly recalled items is scored, with higher scores representing a greater number of story elements recalled. Digit/Number Span Forward and Backward trials measure auditory attention and working memory, respectively. Participants are asked to recall orally presented digits in forward or reverse order, and higher scores represent more digits retained.^27^

##### Score conversion

The NACC neuropsychological battery contains slightly different measures across version changes. Earlier versions included four proprietary measures that were replaced in UDS version 3, with nonproprietary tests assessing the same cognitive domains. The four pairs of tests are highly correlated (*r *= 0.68–0.78), with narrow confidence intervals (CIs) and high prediction accuracy. One study used an equipercentile equating method to produce conversion tables for each pair of tests,^39^ and subsequent research has used these conversions to equate the four pairs of measures across versions.^40^, ^41^ Three of the four pairs of measures were used for this study: BNT and MINT, WMS‐R Logical Memory IIA‐Delayed Recall and Craft Story‐21 Recall‐Delayed, and WMS‐R Digit Span/Number Span. The conversion table provided by Monsell and colleagues^39^ was used to make equivalent scores across versions (e.g., converting Craft Story 21 scores to WMS‐R Logical Memory equivalent scores).

#### 2.3.3 Missing data

**FIGURE 1 alz70361-fig-0001:**
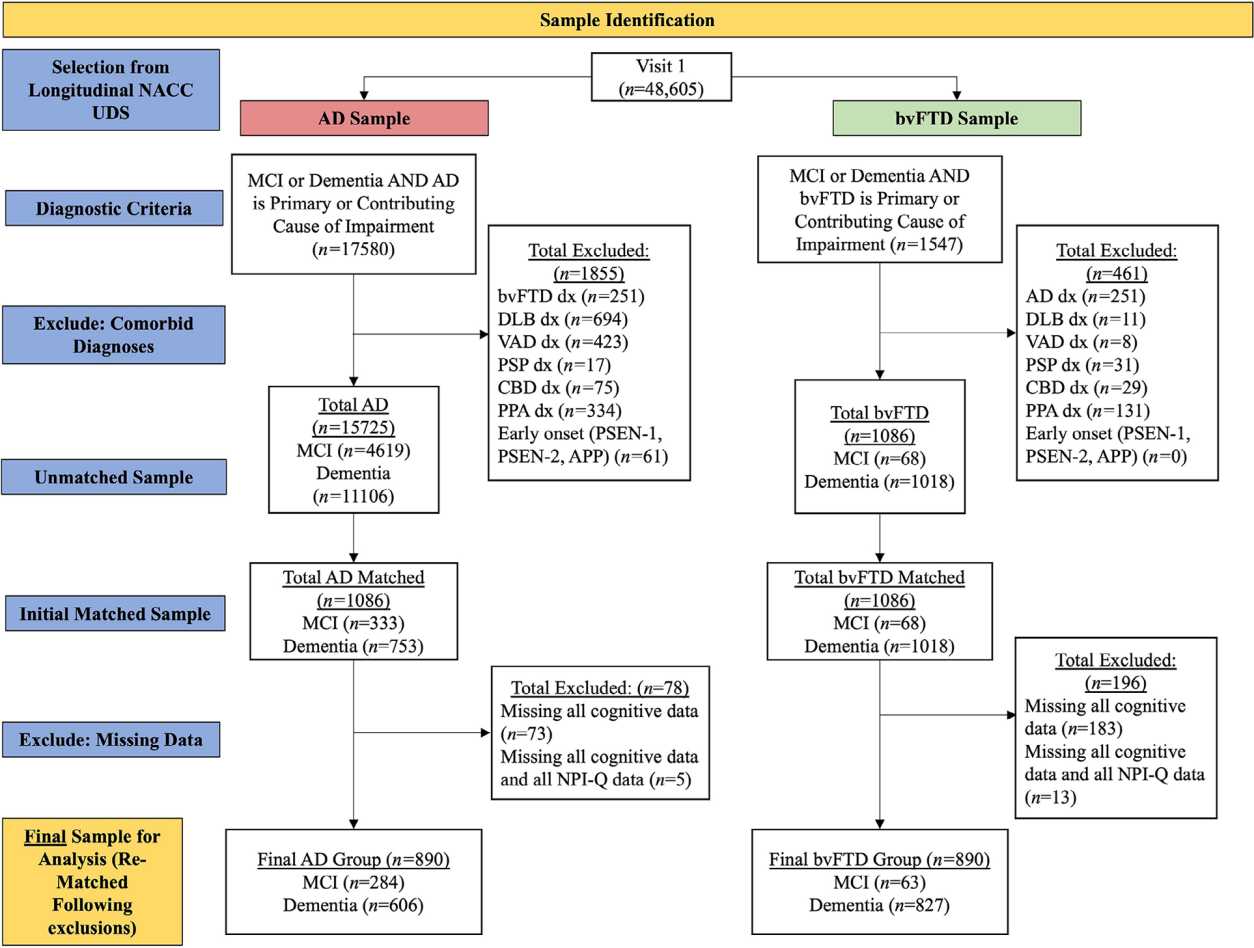
Participant selection diagram. AD, Alzheimer's disease; APP, amyloid precursor protein; bvFTD, behavioral variant frontotemporal dementia; CBD, corticobasal degeneration; VAD, vascular dementia; DLB, Lewy body dementia; MCI, mild cognitive impairment; NACC, National Alzheimer's Coordinating Center; PPA, primary progressive aphasia; PSEN, presenilin; PSP, progressive supranuclear palsy; UDS, Uniform Data Set.

Figure 1 shows the number of participants excluded at each stage. Following initial exclusions, missingness was assessed for both the NPI‐Q and neuropsychological items. Forty‐one participants had missing data across all NPI‐Q variables, 256 participants had missing data across all neuropsychological items, and 18 participants had missing data on both NPI‐Q and neuropsychological items. The UDS forms record several reasons for missing data. Participants who were missing all NPI‐Q items had “Not Available” listed as their reason for missingness, and the majority of participants who were missing all cognitive data had “Not collected due to Cognitive/Behavior Problem” listed as their reason for missingness. There was a somewhat even distribution of AD (47.46%) and bvFTD (52.54%) participants who were missing all NPI‐Q data, whereas there were more participants in the bvFTD group (71.48%) who had missing cognitive data compared to the AD group (28.52%). We excluded participants who were missing both NPI‐Q and cognitive data as well as participants who were missing all cognitive data, as these participants appeared to have more severe impairment (total excluded = 274; 256 missing cognitive data only, 18 missing all data) and did not reflect functioning within early to moderate stages of disease (Table S1). We retained participants who were missing only NPI‐Q data (total retained = 41), as there appeared to be no notable differences in groups on key demographic variables. Following these final exclusions, groups were re‐matched on age, sex, education level, and ethnicity/race. As a robustness check, we generated networks using imputed values, with missing data predicted by a random forest model trained on the observed values. Networks were estimated based on the imputed data. The resulting network was nearly identical to the one generated with participants excluded. However, because the imputed values were difficult to interpret (e.g., a value of 3.4 on the NPI‐Q), we ultimately computed networks with participants excluded from the sample.

### 2.4 Analysis plan

NPS‐COG covariation was modeled with clinical network analysis, a psychometric method for studying the structure of variable associations.^17^, ^18^, ^19^ Within a network perspective, a syndrome is composed of symptom associations that represent complex mechanisms of co‐occurrence. Network analysis provides a graphical representation of observed clinical elements (referred to as “nodes”) and complex direct and indirect associations among them (referred to as “edges”). Network structure (the organization of nodes within a network) and global connectivity (pattern of association between nodes) can provide helpful information about symptom co‐occurrence. Graph metrics can further delineate relative node importance to the overall syndrome network, as nodes may differ in their overall influence on the rest of the network.

#### 2.4.1 Network structure

Analyses were conducted in *R version 4.4.1* using the *qgraph*,^42^ *bootnet*,^43^ *networktools*,^44^ and *NetworkComparisonTest* ^45^ packages. Networks were constructed independently within the bvFTD group and the AD group. Each network consists of 20 nodes that were organized a priori into two clusters or “communities” based on their respective domain. The 12 NPI‐Q items were assigned to the NPS community, and the 8 cognitive subtests were assigned to the COG community. Edges represent partial correlations between variables, and regularization using the graphical least absolute shrinkage and selection operator (GLASSO) algorithm was employed to remove weak and spurious edges.^46^

##### Influential nodes

Nodes with high bridge strength are responsible for connecting node communities^18^, ^43^ and are, therefore, important for understanding comorbidity among symptom domains.^47^ Bridge strength was estimated for each node to identify nodes responsible for inter‐community connectivity (i.e., NPS community–COG community). Bridge strength was calculated by taking the sum of the absolute value of all edges that exist between a given node and its adjacent community.^20^ There is no standard approach to selecting the cutoff for highest bridge strength values; thus, the highest bridge strength nodes are selected based on researcher preference.^20^ We chose a strict cutoff of bridge strength values of *z *> 1, as we believed this would best balance sensitivity and specificity.

#### 2.4.2 Network accuracy

Four aspects of network accuracy were tested to ensure interpretability of the networks: (1) edge‐weight accuracy, (2) centrality stability (CS), (3) edge‐weight and centrality difference tests, and (4) node redundancy.^43^, ^48^ To determine edge‐weight accuracy, nonparametric bootstrapped CIs (95%) were constructed around regularized edge‐weights to determine whether edge‐weights differ significantly.^43^ To determine CS, case‐dropping subset bootstrapping was used to determine the maximum proportion of cases that can be dropped while maintaining a large correlation (*r *= 0.70) between the full‐ and subset‐sample network centrality values.^43^, ^46^ For edge‐weight and centrality difference tests, edge‐weight and node centrality differences were examined using calculated difference scores for each pair of bootstrapped edge‐weight/centrality values.^43^ Finally, goldbricker analysis was conducted to identify node redundancy.^48^

#### 2.4.3 Network comparison tests

The bvFTD and AD networks were compared to determine whether there are significant differences in network metrics across the two clinical groups. Permutation tests were conducted to assess the differences between the two networks based on global strength invariance (i.e., overall connectivity is equivalent across networks) and network structure invariance (i.e., network structure and edge‐weight distribution are equivalent across networks) with false discovery rate (FDR = 0.05) correction.^45^, ^49^

#### 2.4.4 Cross‐sample variability network

In addition to network comparison tests, we computed a network that represents edge‐weight variability between the two networks. We first estimated a cross‐sample network where edge‐weights were averaged across the two networks. We then estimated a cross‐sample variability network where each edge‐weight represents the SD of that edge across the two networks (e.g., edge A‐B = SD of A‐B_AD_ and A‐B_bvFTD_). In other words, edges represent the degree of variability between the two networks, where stronger edges (e.g., greater line thickness) represent greater variability between the two networks.^50^

## 3 RESULTS

### 3.1 Clinical and demographic data

The final sample (*N* = 1780, 890 per group) consisted of older adults (AD: mean age =  63.02, SD = 9.34, 36.4% female; bvFTD: mean age =  62.87, SD = 9.46, 36.52% female) who identified predominantly as non‐Hispanic White (*n* = 1611; 90.5%). The majority met criteria for dementia (AD: 31.91% MCI, 68.09% dementia; bvFTD: 7.08% MCI, 92.92% dementia). The majority of the AD group had Questionable Impairment on the CDR, whereas the majority of the bvFTD group had Mild Impairment on the CDR (Table 1).

**TABLE 1:** Participant demographics by syndrome group.

	AD	bvFTD
*N*	890	890
Age, mean (SD)	63.02 (9.34)	62.87 (9.46)
Sex = Female, n (%)	324 (36.40%)	325 (36.52%)
Education, mean (SD)	15.5 (2.92)	15.36 (3.04)
Ethnicity and Race, n (%)		
Non‐Hispanic White	811 (91.12%)	800 (89.89%)
Hispanic White	21 (2.36%)	23 (2.58%)
Non‐Hispanic Black	14 (1.57%)	18 (2.02%)
Hispanic Black	1 (0.11%)	1 (0.11%)
Other^a^	43 (4.83%)	48 (5.39%)
Cognitive status at visit, n (%)		
MCI	284 (31.91%)	63 (7.08%)
Dementia	606 (68.09%)	827 (92.92%)
CDR Global Impairment Rating, n (%)		
None (0.0)	11 (1.24%)	11 (1.24%)
Questionable (0.5)	512 (57.53%)	280 (31.46%)
Mild (1.0)	305 (34.27%)	411 (46.18%)
Moderate (2.0)	51 (5.73%)	158 (17.75%)
Severe (3.0)	11 (1.24%)	30 (3.37%)

*Note*: Groups are propensity score matched by age, sex, education, and ethnicity/race. Impairment ratings derived from the Clinical Dementia Rating Global Impairment score.

Abbreviations: AD, Alzheimer's disease; bvFTD, behavioral variant frontotemporal dementia; CDR, Clinical Dementia Rating; MCI, mild cognitive impairment; SD, standard deviation.

^a^ Other ethnoracial group includes those identifying as Asian, American Indian/Alaska Native, Native Hawaiian or Other Pacific Islander, Multiracial, and Unknown.

### 3.2 Neuropsychiatric symptoms descriptives

**FIGURE 2 alz70361-fig-0002:**
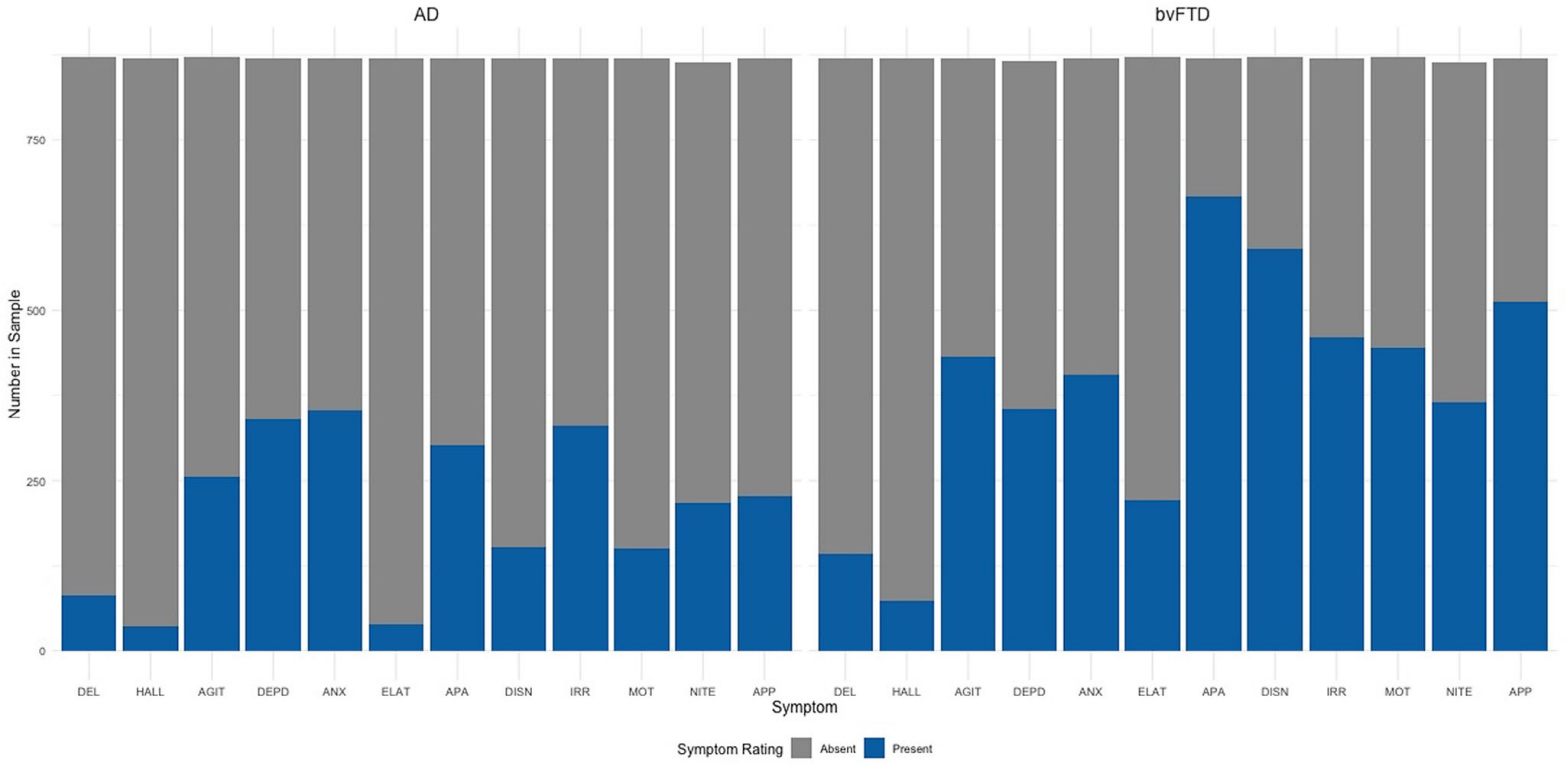
Frequency of endorsed neuropsychiatric symptoms. Frequency of endorsed items on the NPI‐Q at initial visit. Left panel = AD group; right panel = bvFTD group. NPI‐Q = Neuropsychiatric Inventory‐Questionnaire."AD” = Alzheimer's disease. “bvFTD” = behavioral variant frontotemporal dementia. “DEL” = Delusions, “HALL” = hallucinations,“AGIT” = agitation/aggression,“DEP” = depression/dysphoria,“ANX” = anxiety,“ELA” = elation/euphoria,“APA” = apathy/indifference,“DISN” = disinhibition,“IRR” = irritability/lability,“MOT” = motor disturbance”, “NIT” nighttime behaviors, “APP” appetite/eating problems. "NPI‐Q" = Neuropsychiatric Inventory–Questionnaire.

Among those who reported symptoms, NPS severity was generally higher for the bvFTD group (mean = 9.77, SD = 5.86; median number of symptoms endorsed = 5) compared to the AD group (mean = 4.16, SD = 4.12; median number of symptoms endorsed = 2). The chi‐square tests of independence showed that symptom severity differed between groups for each NPI‐Q item, apart from depression/dysphoria (Table 2). For the AD group, anxiety was the most commonly endorsed symptom (40.53%) followed closely by depression/dysphoria (39.08%). For the bvFTD group, apathy/indifference (76.58%) was the most commonly endorsed symptom (Figure 2).

**TABLE 2:** Frequency of neuropsychiatric symptom severity ratings by group.

	No symptoms	Mild	Moderate	Severe
Delusions^**^				
AD	90.60%	5.16%	3.33%	0.92%
bvFTD	83.70%	7.23%	6.32%	2.76%
Hallucinations^*^				
AD	95.87%	2.87%	1.15%	0.12%
bvFTD	91.49%	4.71%	3.22%	0.58%
Agitation/Aggression^**^				
AD	70.84%	19.29%	7.46%	2.41%
bvFTD	50.40%	21.70%	21.47%	6.43%
Depression/Dysphoria				
AD	60.92%	24.71%	11.72%	2.64%
bvFTD	59.05%	22.26%	16.03%	2.65%
Anxiety^**^				
AD	59.47%	25.37%	13.09%	2.07%
bvFTD	53.45%	20.23%	21.03%	5.29%
Elation/Euphoria^**^				
AD	95.52%	2.53%	1.84%	0.12%
bvFTD	74.54%	9.40%	12.73%	3.33%
Apathy/Indifference^**^				
AD	65.44%	19.29%	11.60%	3.67%
bvFTD	23.45%	20.46%	33.56%	22.53%
Disinhibition^**^				
AD	82.43%	11.25%	5.51%	0.80%
bvFTD	32.26%	22.73%	27.33%	17.68%
Irritability/Lability^**^				
AD	62.00%	24.34%	11.48%	2.18%
bvFTD	47.13%	21.26%	19.77%	11.84%
Motor Disturbance^**^				
AD	82.64%	10.46%	4.94%	1.95%
bvFTD	48.85%	16.28%	20.76%	14.11%
Nighttime Behaviors^**^				
AD	74.88%	14.12%	8.68%	2.32%
bvFTD	57.92%	16.53%	18.04%	7.51%
Appetite/Eating problems^**^				
AD	74.02%	16.32%	8.16%	1.49%
bvFTD	41.22%	18.71%	27.67%	12.40%

*Note*: Aggregate neuropsychiatric symptom ratings derived from the Neuropsychiatric Inventory–Questionnaire (NPI‐Q). Item‐level ratings: no symptoms = 0, mild = 1, moderate = 2, severe = 3.

Abbreviations: AD, Alzheimer's disease; bvFTD, behavioral variant frontotemporal dementia.

* Groups differed at *p* < 0.05.

** Groups differed at *p* < 0.001.

### 3.3 Differences in cognitive performance

The AD group performed significantly better than the bvFTD group on Category Fluency (AD: mean = 21.71, SD = 9.96; bvFTD: mean = 18.19, SD = 10.49; *p *< 0.001, *d* = 0.34), Naming (AD: mean = 23.82, SD = 6.28; bvFTD: mean = 21.77, SD = 7.59; *p *< 0.001, *d *= 0.29), and Digits Backward (AD: mean = 4.60, SD = 2.33; bvFTD: mean = 4.32, SD = 2.43; *p *< 0.05, *d *= 0.12). The bvFTD group scored significantly better than the AD group on story memory (AD: mean = 3.88, SD = 4.48, bvFTD: mean = 6.04, SD = 5.49; *p *< 0.001, *d *= 0.43). Groups did not differ on TMT‐A, TMT‐B, Digit Symbol, Digits Forward, or Global Cognition (Table 3).

**TABLE 3 alz70361-tbl-0003:** Differences in cognitive scores between AD and bvFTD.

	AD	bvFTD	
	Mean (SD)	Mean (SD)	*d*
Trail Making Test Part A	59.83 (40.13)	59.14 (37.05)	0.02
Trail Making Test Part B	164.58 (94.01)	163.31 (93.12)	0.01
WAIS‐R Digit Symbol	30.16 (16.19)	31.63 (14.79)	0.09
Category Fluency	21.71 (9.96)	18.19 (10.49)	0.34^**^
Naming	23.82 (6.28)	21.77 (7.59)	0.29^**^
Story Memory	3.88 (4.48)	6.04 (5.49)	0.43^**^
Digits Forward	7.07 (2.47)	6.90 (2.46)	0.07
Digits Backward	4.60 (2.33)	4.32 (2.43)	0.12^*^
Global Cognition	23.01 (5.86)	23.29 (5.94)	0.05

*Note*: Means, standard deviations (SDs), and effect sizes of cognitive scores between the AD and bvFTD groups. Category Fluency represents the total score on two semantic categories. Naming, Story Memory, Digits Forward, and Digits Backward were calculated using the NACC equipercentile equating method between test versions within that category (e.g., Naming = Boston Naming Test/Multilingual Naming Test; Story Memory = WMS‐R Logical Memory IIA‐Delayed Recall/Craft Story‐21 Recall‐Delayed; Digits Forward = Digit Span Forward/Number Span Forward; Digits Backward = Digit Span Backward/Number Span Backward); Global Cognition = MoCA/MMSE.

Abbreviations: AD, Alzheimer's disease; bvFTD, behavioral variant frontotemporal dementia; SD, standard deviation. *d* = Cohen's *d* measure of effect size.

* Group means differed at *p* < 0.05.

** Group means differed at *p* < 0.001.

### 3.4 Network accuracy

Network accuracy test results suggested that both networks were stable and interpretable.

#### 3.4.1 Edge‐weight accuracy

Nonparametric bootstrapped CIs were tight around the parameter estimates for edge‐weights for both networks, suggesting that the edge‐weight value estimations are interpretable (Figure S1).

#### 3.4.2 Centrality stability

Bridge strength was stable under subsetting cases in both the AD network [*CS*(cor = 0.7) = 0.67] and in the bvFTD network [*CS*(cor = 0.7) = 0.75] (Figure S2).

#### 3.4.3 Difference tests

In both networks, bootstrapped difference tests showed that edge‐weight values and bridge strength values differed significantly from one another, suggesting that the order of edge‐weights and bridge strength values are interpretable (Figures S3 and S4).

#### 3.4.4 Node redundancy

Goldbricker analysis identified potential colinear nodes in the AD network (TMT‐A and TMT‐B), and in the bvFTD network (TMT‐A and TMT‐B; irritability/lability and agitation/aggression; digits backward and digits forward; and digits backward and category fluency). Collinearity is expected given the high correlation among neuropsychological tests and especially those tests assessing related domains/constructs through similar methods.^51^ However, combining the node pairs would result in loss of important clinical information, so each node is retained in both networks and findings are interpreted with caution.

### 3.5 Network structure

**FIGURE 3 alz70361-fig-0003:**
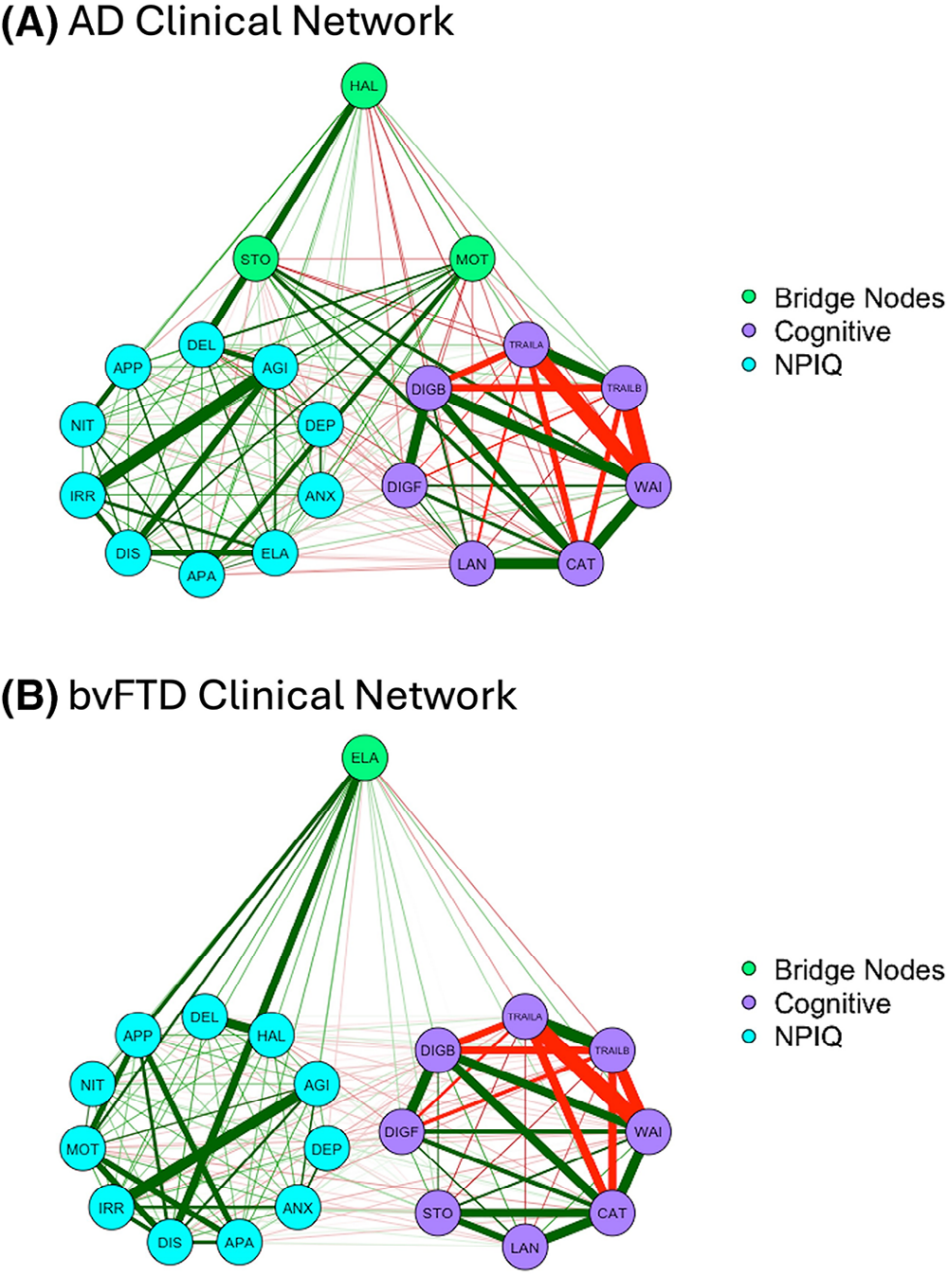
Clinical networks in AD and bvFTD. Clinical networks for the AD group (A) and bvFTD group (B). Nodes were assigned to two communities. NPS nodes are in blue. COG nodes are in purple. Nodes with highest bridge strength are light green. Green lines represent positive edges, and red lines represent negative edges. Line thickness represents the strength of the statistical relationship. “TRAILA” = Trail Making Test Part A, “TRAILB” = Trail Making Test Part B, “WAI” = WAIS‐R Digit Symbol, “CAT” = Category Fluency, “LAN” = Boston Naming Test/Multilingual Naming Test, “STO” = Logical Memory/Craft Story‐21, “DIGF” = Digit span/Number Span Test forward, “DIGB” = Digit Span/Number Span Test Backward. "NPIQ" = Neuropsychiatric Inventory–Questionnaire.

Both the AD and bvFTD networks were densely connected within and between NPS and COG communities, consisting of both positive and negative edges (Figure 3). Edges among NPS variables were positive, suggesting that NPS severity in one symptom was associated with increased severity in other symptoms. Edges among cognitive variables were positive and negative, consistent with anticipated patterns between cognitive domains and the test scoring (e.g., greater time to completion on TMT‐A was associated with fewer items completed on digit symbol). Edges connecting NPS and COG nodes were largely negative, suggesting that increased NPS severity was associated with worse COG performance. Both networks are discussed separately, below.

#### 3.5.1 AD network structure

In the AD network, 83 of 190 edges (43.7%) were retained following regularization (mean edge‐weight = 0.022). The strongest edges within the NPS community included agitation/aggression and irritability/lability (edge‐weight = 0.37), nighttime behaviors and appetite/eating problems (edge‐weight = 0.21), and depression/dysphoria and anxiety (edge‐weight = 0.20). The strongest edges within the COG community included naming and category fluency (edge‐weight = 0.40), digits forward and digits backward (edge‐weight = 0.38), digit symbol and TMT‐A (edge‐weight = ‐0.37), and digit symbol and TMT‐B (edge‐weight = ‐0.47). The strongest (albeit small) inter‐community connections were found between motor disturbance and digits backward (edge‐weight = ‐0.05), delusions and category fluency (edge‐weight = ‐0.03), and hallucinations and naming (edge‐weight = –0.31).

#### 3.5.2 AD network bridge strength

**FIGURE 4 alz70361-fig-0004:**
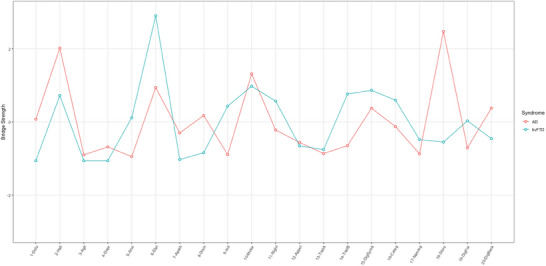
Bridge strength values for the AD and bvFTD Networks. Standardized bridge strength values (*z* scores) for all 20 clinical nodes across both syndrome groups. See Figures 2 and 3 for full variable names.

Story memory (*z *= 2.47), hallucinations (*z *= 2.02), and motor disturbance (*z* = 1.31) had the highest bridge strength, suggesting that they served as bridge connections between the NPS and COG communities (Figure 4).[Fig alz70361-fig-0002]

#### 3.5.3 bvFTD network structure

In the bvFTD network, 67 of 190 edges (35%) were retained following regularization ( mean edge‐weight = 0.023). The strongest edges within the NPS community included agitation/aggression and irritability/lability (edge‐weight = 0.38), hallucinations and delusions (edge‐weight = 0.25), apathy/indifference and appetite/eating problems (edge‐weight = 0.22), and elation/euphoria and disinhibition (edge‐weight = 0.21). The strongest edges within the COG community included digits forward and digits backward (edge‐weight = 0.36), naming and category fluency (edge‐weight = 0.29), story memory and category fluency (edge‐weight = 0.28), TMT‐A and TMT‐B (edge‐weight = 0.23), story memory and naming (edge‐weight = 0.22), and digit symbol and TMT‐A (edge‐weight = ‐0.45). The strongest inter‐community connections were found between elation/euphoria and TMT‐B (edge‐weight = –0.03) and motor disturbance and category fluency (edge‐weight = –0.02), although edges were small.

#### 3.5.4 bvFTD network bridge strength

Elation/euphoria (*z* = 2.90) had the highest bridge strength, suggesting that this symptom served as a bridge connection between NPS and COG communities (Figure 4). This was followed by motor disturbance (*z *= 0.98), digit symbol (*z* = 0.86), and TMT‐B (*z *= 0.76); however, these did not reach our *z *> 1 threshold.

### 3.6 Network comparisons

Network comparison test results showed that the AD and bvFTD networks did not significantly differ in global strength (*S *= 0.42, *p* = 0.37), where *S* equals the difference in global strength between the two groups. In other words, there were no differences in the level of connectivity or density between the AD and bvFTD networks. The omnibus test of network structure invariance showed that the two networks had significantly different network structures (*M* = 0.25, *p* = 0.003), where *M* equals the maximum difference in edge‐weights between the two networks. In other words, the AD and bvFTD networks have distinct patterns of connectivity in this sample. Post hoc comparisons showed significant differences in four edges between the two networks. In the AD network, edge‐weights were stronger (in the positive or negative direction) than the bvFTD network for the following node pairs: (1) depression and apathy/indifference, (2) disinhibition and nighttime behaviors, (3) delusions and motor symptoms, (4) appetite/eating problems and story memory, and (5) TMT‐B and digit symbol. In the bvFTD network, the edge‐weight connecting naming and story memory was stronger compared to the AD network.

In the cross‐sample variability network, the most variable edges between the AD and bvFTD networks were between TMT‐B and digit symbol (SD = 0.19), story memory and naming (SD = 0.16), motor disturbance and anxiety (SD = 0.13), and story memory and hallucinations (SD = 0.13) (Figure S5).[Table alz70361-tbl-0003]

## 4 DISCUSSION

Nuanced understanding of common and distinct clinical mechanisms is important for precise characterization of ADRD. Characterization of AD and bvFTD is difficult due to overlapping clinical presentations.^12^, ^14^ The present study used network methods to compare cognitive and neuropsychiatric functioning between AD and bvFTD. AD and bvFTD showed distinct clinical network structures as well as distinct influential symptoms responsible for overall network maintenance. These differences identify novel clinical features of AD and bvFTD that previous methods have not identified and may assist in identifying patient‐specific intervention targets.

The unique features of the NACC database and the ADRC program made this investigation possible. NACC offers an accessible approach to clinical research, particularly for institutions that would not otherwise have access to neuropsychological, biological, or neuroimaging data. Participants in this sample are well phenotyped clinically, allowing for precise investigations with generalizable results. In addition, item‐level and domain‐level neuropsychiatric and neurocognitive data improve the nuanced characterization possible through network analysis.

### 4.1 Distinct clinical network structures in AD and bvFTD

The most novel aspect of our study was the statistical comparison of clinical networks between AD and bvFTD. There was a statistically significant difference in network structure between AD and bvFTD, and differences in edge‐weights were found within network communities. This is further demonstrated in the cross‐sample variability network, which highlights structural differences between the AD and bvFTD networks. This is consistent with research suggesting that patterns of comorbidity, rather than symptom severity or presence of isolated symptoms, are more useful for clinical characterization of ADRD syndromes.^52^ In contrast, global strength (e.g., overall level of connectivity) did not differ significantly between the two groups, possibly pointing to similar levels of symptom comorbidity. Overall, our findings reveal unique clinical network phenotypes in AD and bvFTD at initial clinic visit, highlighting the potential of network analysis for precise syndrome characterization.[Fig alz70361-fig-0003], [Fig alz70361-fig-0004]

### 4.2 Neuropsychiatric symptom burden and cognitive performance

Patients with bvFTD had greater NPS burden compared to patients with AD, consistent with clinical presentations at initial visit.^53^, ^54^ In the AD group, anxiety and depression/dysphoria were the most frequently endorsed items on the NPI‐Q, whereas apathy/indifference was the most frequently endorsed item in the bvFTD group. Mood symptoms are common in AD and are often present early in the disease course.^21^, ^55^, ^56^ Apathy is common in both bvFTD^9^, ^57^, ^58^ and AD,^59^, ^60^ and it is present during the prodrome of both conditions, further obscuring differential diagnosis. The groups performed similarly on cognitive testing, and where groups differed, effect sizes were small, highlighting the unique contribution of a network perspective in detecting differences in clinical functioning between groups. As expected, the bvFTD group outperformed the AD group on story memory, which is consistent with the hallmark amnestic neuropsychological profile in AD^61^ and relative sparing of memory functions in bvFTD.^9^ The AD group outperformed the bvFTD group on Digits Backward, possibly representing greater working memory deficits among patients with bvFTD.

### 4.3 Comorbidity among and between clinical domains

There was strong intra‐community connectivity in both the AD and bvFTD networks. Consistent with previously identified NPS clinical networks in ADRD,^21^ the NPS community was characterized by positive edges, representing NPS comorbidity in both conditions. COG nodes were also highly connected, likely representing overlapping cognitive processes among tasks (e.g., processing speed in Digit Symbol and TMT‐A).^28^ NPS and COG were interrelated, as evidenced by dense inter‐community connections in both groups. Inter‐community edge‐weights were small, although the pattern of associations is clinically meaningful. Consistent with previous research,^58^, ^62^, ^63^ inter‐community edges were largely negative, where increased NPS severity was associated with reduced performance across cognitive tasks. Networks highlighted potentially frontally‐mediated mechanisms in AD (e.g., digits backward and motor disturbance, or perseverative and stereotypic motor behaviors) and bvFTD (e.g., elation/euphoria and TMT‐B).^64^ Other patterns of associations were less intuitive (e.g., motor dysfunction and category fluency in bvFTD) and may warrant further investigation.

### 4.4 Influential bridge symptoms in AD and bvFTD

In the AD network, story memory performance had the highest bridge strength, serving as a connector between NPS and COG. AD is typically characterized by an amnestic presentation at early stages, and a substantial body of research describes the association between memory decline and NPS in AD.^65^, ^66^ Previous research has also demonstrated the prominent role of episodic memory performance within cognitive networks^67^, ^68^, ^69^ and between COG and NPS network communities^70^ in AD. Our results further extend these findings, emphasizing the role of memory performance in symptom comorbidity in AD. Of interest, although less common in our sample, hallucinations and motor disturbance also emerged as bridge symptoms between the NPS and COG communities. This finding may be an artifact of disease progression, although both symptoms may occur at any stage of illness.^4^

Elation/euphoria was the only bridge symptom above the threshold that connected NPS and COG in the bvFTD group. Elation/euphoria (or excessive happiness and gregariousness) is a core feature of bvFTD and reflects significant behavioral change.^10^, ^57^ Compared to AD, elation/euphoria tends to be more common and severe in bvFTD,^71^, ^72^ likely due to frontal network dysfunction.^64^ The association between elation and neurocognitive function is unclear. In cognitively normal older adults, elation/euphoria was associated with reduced processing speed and attention,^73^ whereas in patients with bvFTD, euphoria was not associated with cognitive performance.^74^ Nevertheless, elation/euphoria may present a novel treatment target that would both reduce NPS and limit the impact of NPS on COG.

### 4.5 Implications for research and practice

The present study has several implications for research and clinical practice. First, NPS and COG were highly interrelated in both networks, modeling complex relationships between these domains that align with the symptom complexity observed clinically. “Activation” or presence of one symptom results in cascading activation of other symptoms throughout a syndrome network. Clinical network analysis may be useful for predicting symptom development of phenotypic profiles, as certain clinical features (e.g., elation/euphoria) may herald the onset or co‐occurrence of other related symptoms (e.g., disinhibition) not directly observed during evaluation. Clinical networks elucidate those symptoms that are strongly connected. In addition, benefits of intervention may be maximized if certain symptoms or symptom clusters are targeted, as this may result in overall symptom alleviation. Second, targeting bridge symptoms may be an efficient approach to minimizing comorbidity.^18^, ^20^, ^43^ Computer‐simulated “treatment” of bridge nodes has been shown to reduce network connectivity (or “contagion”) between symptom communities,^18^, ^20^ offering an innovative and cost‐effective approach to clinical trial design. Third, distinct patterns of AD and bvFTD clinical networks suggest unique patterns of comorbidity. The clinical networks described in this study may enable more precise symptom characterization and monitoring in both clinical practice and research (e.g., clinical trials). Finally, the above analyses may provide an additional reference point for comparison of future bvFTD biomarkers. Clinical network phenotypes may provide insight into biomarker targets in bvFTD and may provide a blueprint for identifying neuroanatomic substrates of neuropsychiatric and cognitive change as candidate targets advance.

### 4.6 Limitations

This study used a convenience sample, consisting primarily of non‐Hispanic White patients seeking memory care in urban academic centers. In addition, most AD samples have more women than men, reflecting the greater prevalence of AD among women, whereas the present sample has fewer women compared to men. Thus, generalizability of these results remains uncertain. Networks were estimated based on cross‐sectional data, which limits our ability to make causal inferences about node relationships. Future studies comparing clinical networks over time may offer valuable insights into distinct patterns of clinical progression in AD and bvFTD. As an initial investigation, participants were identified based on syndrome classification with strict sample selection procedures to reduce possible influence of comorbid neurodegenerative conditions. However, analyses should be replicated with a pathologically verified sample to confirm diagnostic status and to assess the robustness of the network‐based approach for syndrome and etiologic characterization. Future research examining the use of initial visit clinical network features for prediction of later autopsy‐confirmed diagnoses will be crucial for clinical application. In addition, bvFTD can be misdiagnosed as a primary psychiatric disorder due to overlapping clinical symptoms,^75^ and future research comparing clinical network phenotypes between these conditions is warranted.

Although participants were matched on age, sex, education, and race/ethnicity, we opted to not match based on cognitive status or symptom severity to more accurately reflect base rates and symptom presentations at initial clinical visit. Previous research highlights that patterns of clinical functioning are better able to classify ADRD syndromes compared to overall severity (e.g., CDR).^52^ Furthermore, disease severity is not commonly considered in bvFTD diagnosis. Neurocognitive networks have been shown to be similar across severity levels (e.g., MCI and dementia) in AD samples,^67^, ^69^ although future work could strengthen the evidence base by matching on severity. In addition, informant‐reported symptoms are subject to bias, particularly when symptoms are severe and distressing. The NPI‐Q asks about symptoms within the last month, which may miss important information about fluctuating symptom presentation. Additional informants would reduce bias and provide a more accurate depiction of symptom course. Finally, racial and ethnic differences in clinical presentations in ADRD are understudied and should be prioritized in future work to ensure that findings are generalizable and accessible to diverse populations.

## 5 SUMMARY

In summary, the present study highlights a novel approach for precise syndrome characterization and offers unique insights into unique patterns of clinical functioning in AD and bvFTD. Findings may advance characterization at initial clinic visit and contribute to solving urgent diagnostic challenges in bvFTD.

## CONFLICT OF INTEREST STATEMENT

Jeffrey L. Cummings has provided consultation to Acadia, Acumen, ALZpath, Annovis, Aprinoia, Artery, Biogen, Biohaven, BioXcel, Bristol‐Myers Squib, Eisai, Fosun, GAP Foundation, Green Valley, Janssen, Karuna, Kinoxis, Lighthouse, Lilly, Lundbeck, LSP/eqt, Mangrove Therapeutics, Merck, MoCA Cognition, New Amsterdam, Novo Nordisk, Optoceutics, Otsuka, Oxford Brain Diagnostics, Praxis, Prothena, ReMYND, Roche, Scottish Brain Sciences, Signant Health, Simcere, sinaptica, T‐Neuro, TrueBinding, and Vaxxinity pharmaceutical, assessment, and investment companies. Jeffrey L. Cummings owns the copyright of the Neuropsychiatric Inventory. The rest of the authors (Grace J. Goodwin, Sebastian Mehrzad, Brenna N. Renn, Jefferson W. Kinney, and Samantha E. John) have no conflicts to disclose. Author disclosures are available in the supporting information.

## Supporting information



Supporting Information

Supporting Information
