# Supplementary material for: Comparing clinical features of behavioral variant frontotemporal dementia and Alzheimer's disease using network analysis
Source: Alzheimers Dement. 2025 Jun 17;21(6):e70361. doi: 10.1002/alz.70361 (PMC12173837; doi:10.1002/alz.70361)
Supplement: Supplementary file 2 — Supporting Information [file ALZ-21-e70361-s001.docx]

**Supplementary Materials**

Supplementary Table 1. Demographics of Participants with Missing NPI-Q or Cognitive Data

|  | **Missing NPI-Q Data Only**  **(n = 41)** | **Missing Cognitive Data Only**  **(n = 256)** | **Missing All NPI-Q and Cognitive Data**  **(n = 18)** |
| --- | --- | --- | --- |
| Age (Mean/SD) | 63.27(9.65) | 61.24(10.6) | 66.56(8.51) |
| Sex (% female) | 90.24% | 42.97% | 72.22% |
| Education (Mean/SD) | 15.32(2.86) | 14.73(3.5) | 16.65(2.62) |
| Ethnicity/Race (%) |  |  |  |
| Non-Hispanic White | 90.24% | 82.03% | 72.22% |
| Hispanic White | 0.00% | 6.64% | 0.00% |
| Non-Hispanic Black | 2.44% | 2.73% | 0.00% |
| Hispanic Black | 0.00% | 0.39% | 0.00% |
| Other^*^ | 7.32% | 8.20% | 27.78% |
| Diagnosis (%) |  |  |  |
| AD | 56.10% | 28.52% | 27.78% |
| bvFTD | 43.90% | 71.48% | 72.22% |
| CDR (%) |  |  |  |
| None (0.0) | 0.00% | 0.00% | 0.00% |
| Questionable (0.5) | 63.41% | 6.25% | 0.00% |
| Mild (1.0) | 21.95% | 19.14% | 16.67% |
| Moderate (2.0) | 12.20% | 32.03% | 5.56% |
| Severe (3.0) | 2.44% | 42.58% | 77.78% |

*Note.* Impairment ratings derived from the Clinical Dementia Rating Global Impairment score. AD = Alzheimer’s disease. bvFTD = behavioral variant frontotemporal dementia. SD = standard deviation. CDR = clinical dementia rating. *Other ethnoracial group includes those identifying as Asian, American Indian/Alaska Native, Native Hawaiian or Other Pacific Islander, Multiracial, and Unknown. Patients missing NPI-Q data only were retained in the sample.

Supplementary Figure 1. Bootstrapped Confidence Intervals of Estimated Edge-Weights


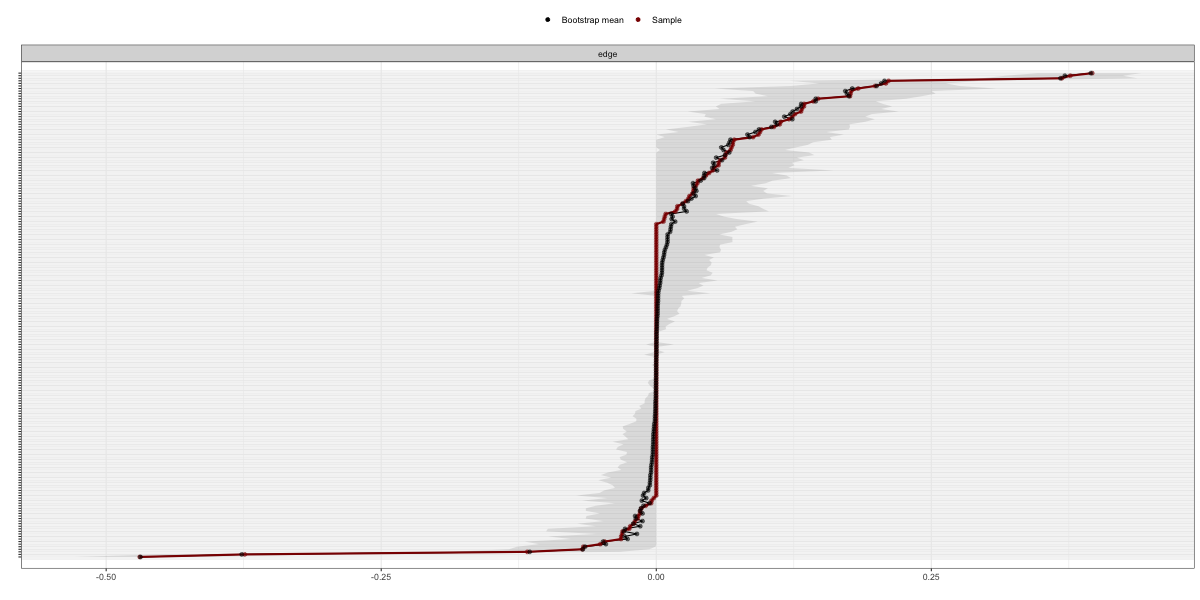


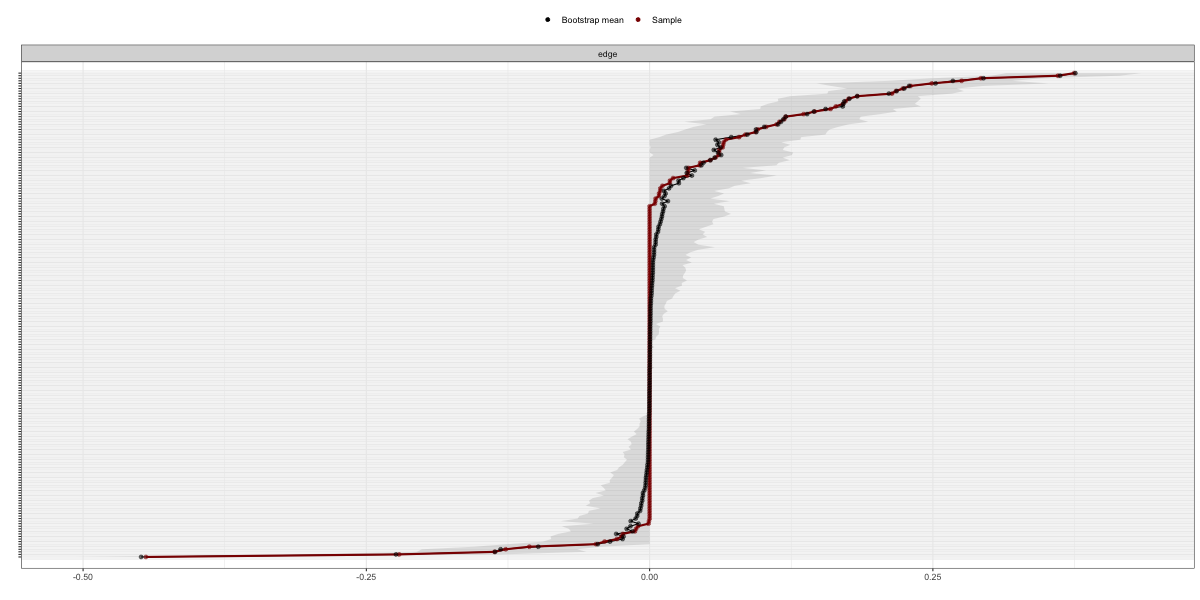


*Note.* Top: AD network. Bottom: bvFTD network. Red lines represent sample values. Gray lines represent bootstrapped confidence intervals.

Supplementary Figure 2. Centrality Stability Across Subsetting Cases.


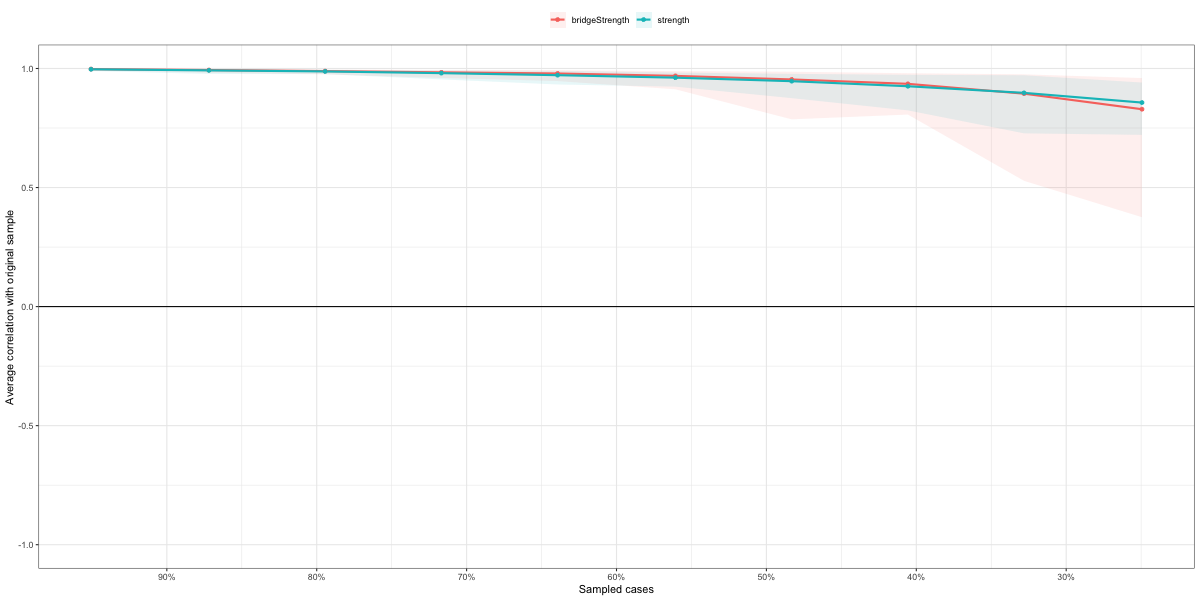


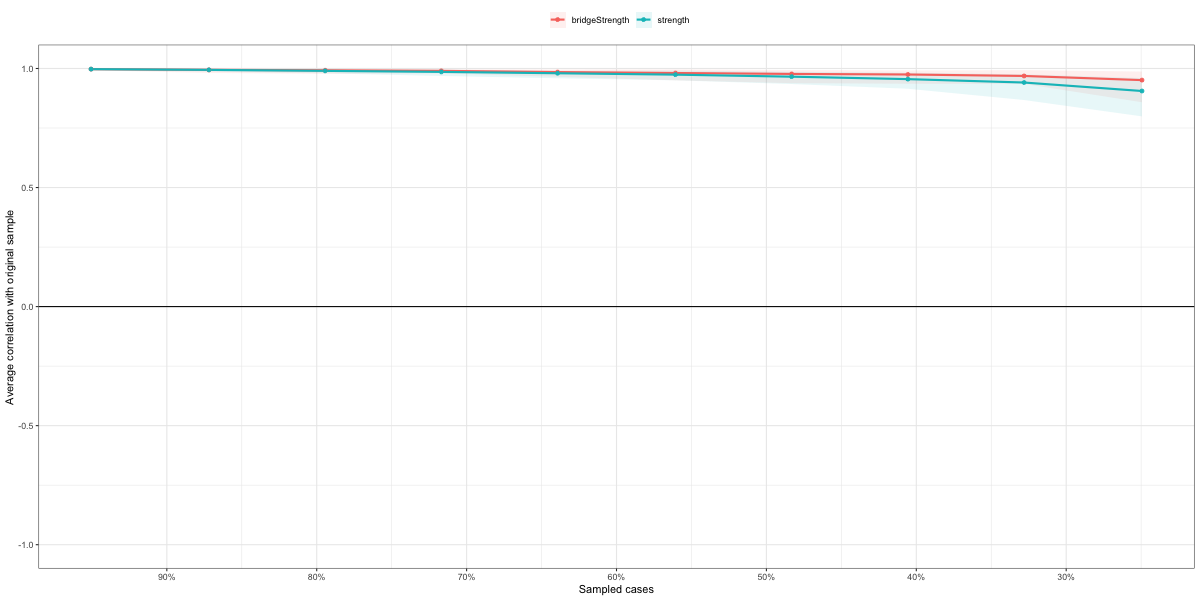


*Note.* Average Correlations Between Bridge Strength of Original Network and Networks Sampled with Persons Dropped. Top: AD network. Bottom: bvFTD network.

Supplementary Figure 3. Bootstrapped Difference Tests Between Non-Zero Edge-Weights


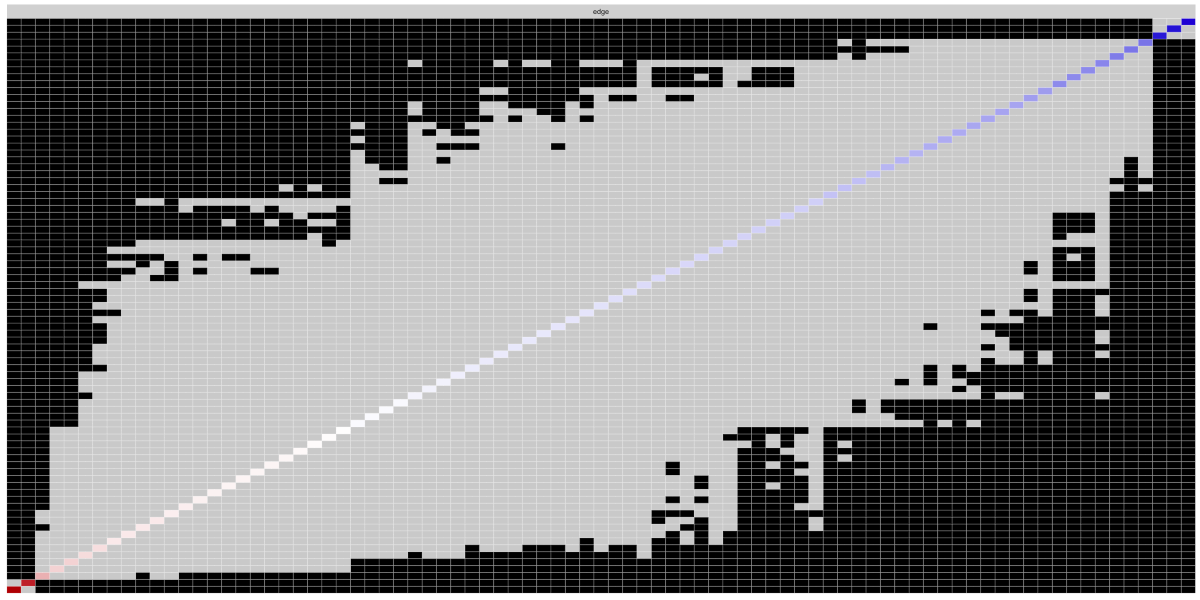


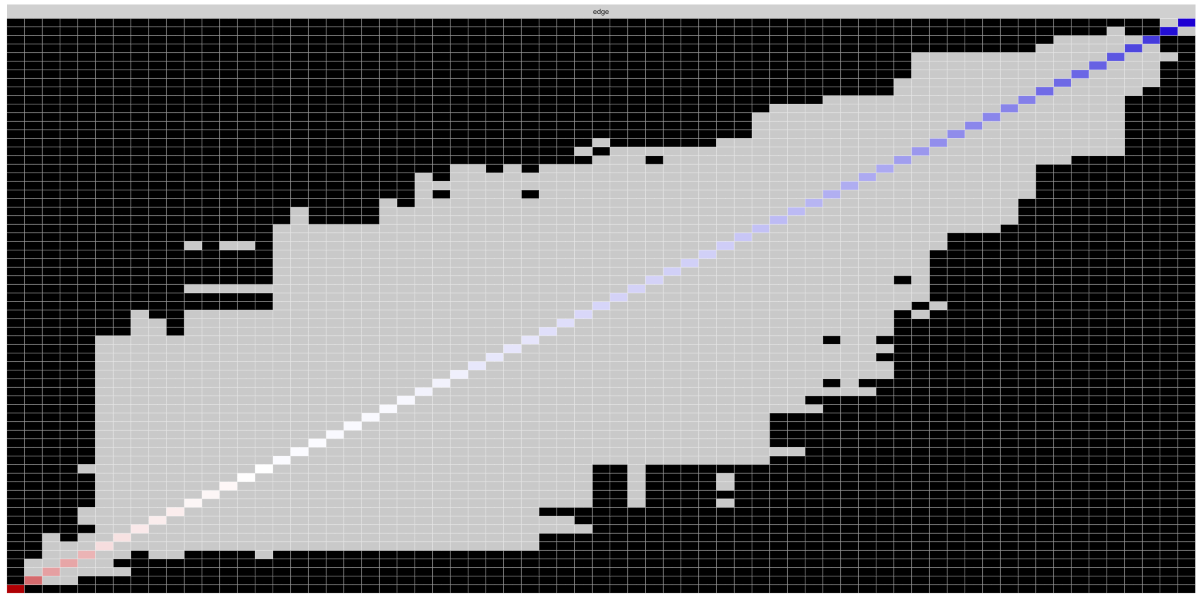


*Note.* Top: AD network. Bottom: bvFTD network. Each node pair is represented on both axes. Black boxes represent edges that significantly differ from one another. Gray boxes represent edges that do not significantly differ from one-another. Colored boxes on the diagonal correspond to the color of the edge in Figure 3.

Supplementary Figure 4. Bootstrapped Difference Tests Between Bridge Strength Values


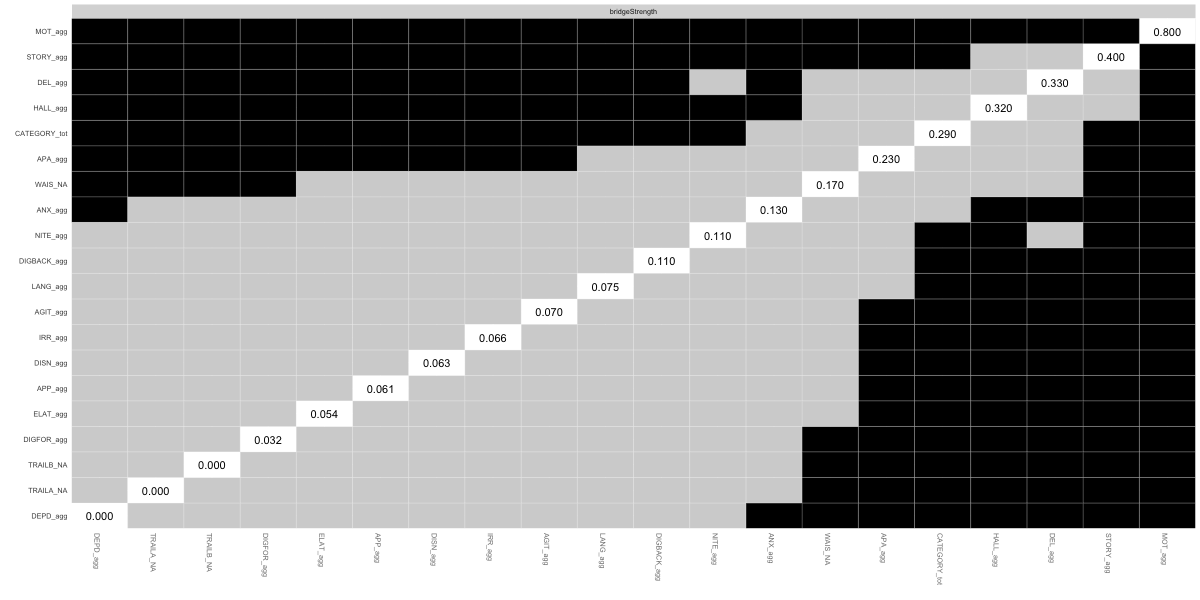


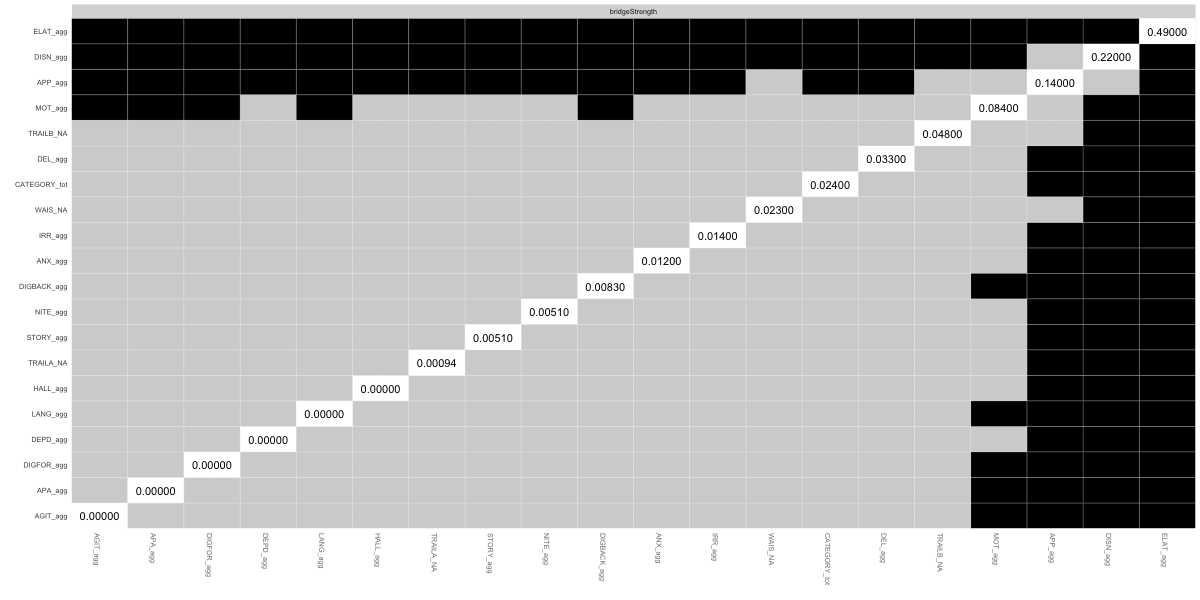


*Note.* Top: AD network. Bottom: bvFTD network. Each node is represented once on the X axis and once on the Y axis. Black boxes represent nodes that significantly differ in bridge strength. Gray boxes represent nodes that do not significantly differ in bridge strength. White boxes on the diagonal show the value of bridge strength.

Supplementary Figure 5. Cross-Sample Variability Network


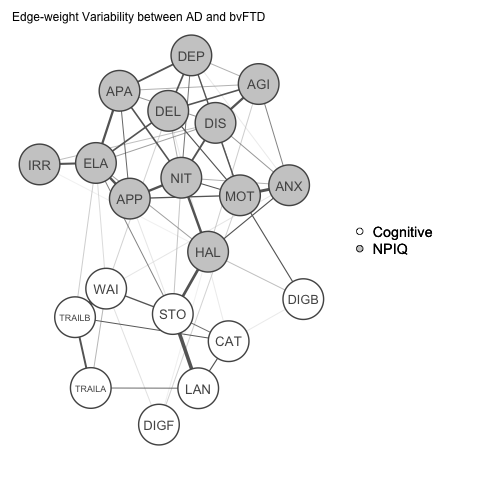


*Note.* Each edge in the network represents the standard deviation of this edge between the AD and bvFTD networks. Thicker lines represent greater edge-weight variability between the two networks. See Figures 2-3 for defined abbreviations.
